# From Hemp Waste to Bioactive Nanofiber Composites: Deep Eutectic Solvents and Electrospinning in Upcycling Endeavors

**DOI:** 10.3390/gels10010001

**Published:** 2023-12-19

**Authors:** Cláudia Mouro, Ana P. Gomes, Isabel C. Gouveia

**Affiliations:** FibEnTech Research Unit, Faculty of Engineering, University of Beira Interior, 6200-001 Covilhã, Portugal; claudia.mouro@ubi.pt (C.M.); anapaula@ubi.pt (A.P.G.)

**Keywords:** hemp, agro-waste, deep eutectic solvents, electrospinning, gel-based blends, waste valorization, nanocomposite gels, sustainable materials

## Abstract

Natural fibers have attracted increasing interest as an alternative to produce environmentally friendly and sustainable materials. Particularly, hemp fibers have been widely used in various industrial applications due to their extremely unique properties. However, hemp can generate a large amount of agro-waste, and it results in an attractive source of biopolymers for the development of low-cost materials as an alternative to the raw materials and conventional petroleum-based plastics. In addition, deep eutectic solvents (DESs), a new type of truly green solvents, have been shown to remove gums, lignin, and other non-cellulosic components from hemp fibers. Reusing these components dissolved into the DESs to fabricate new materials directly by electrospinning is a very attractive but still unexplored endeavor. Thus, this innovative research to venture new upcycling pathways is focused on the fabrication of composite nanofibers by electrospinning of a gel-based blend of Poly(vinyl alcohol) (PVA) and hemp agro-waste (HW) dissolved into choline chloride (ChCl):Glycerol (1:2) and ChCl:Urea (1:2) DES mixtures. The results obtained revealed that the produced nanofibers displayed uniform appearance with diameters ranging from 257.7 ± 65.6 nm to 380.8 ± 134.0 nm. In addition, the mechanical properties of the electrospun composite nanofibers produced from the gel-based blends of HW dissolved in DESs and PVA (HW-DESs_PVA) were found to be superior, resulting in an enhanced tensile strength and Young’s modulus. Furthermore, the incorporation of HW into the nanofibers was able to provide bioactive antioxidant and antibacterial properties. Overall, this study demonstrated a promising, more sustainable, and eco-friendly way to produce electrospun composite nanofibers using HW in a circular economy perspective.

## 1. Introduction

The use of natural fibers from plants has attracted increasing interest in recent years due to their low cost, abundance, biodegradability, low density, high stiffness, and strength-to-weight ratio [1,2,3,4]. In addition, among the different natural fibers, hemp fiber has engaged a prominent position on the international scene due to its advantages both in terms of cultivation and application [2,5,6,7].

Hemp has the ability to grow rapidly under mild and harsh climate conditions throughout the world [1]. Moreover, the hemp planting process does not require the use of fertilizers, herbicides, and insecticides, requires 50% less water consumption than the production and processing of cotton, and it is possible to obtain a production yield 250 times greater per unit area [5,8,9]. Furthermore, hemp can help refresh depleted soil by restoring stability and nutrients to the area and presents the potential to absorb contaminants [6,10]. On the other hand, in terms of industrial applications, hemp fibers have proven to be a source of green and sustainable raw materials and products for use in textiles, paper, food, medicine, personal care products, animal feed, biofuel, automotive accessories, building materials, and chemical feedstocks for paints, biodegradable plastics, and adhesives due to their high tenacity, high softness, great sweat-wicking performance, antibacterial capability, exceptional UV and corrosion resistance ability, among other extraordinary properties [1,6,8,9,11,12,13]. Nevertheless, these plant fibers are highly prone to wrinkling, display a hydrophilic character, and are susceptible to contamination and microbial attack. Hence, there is a need for approaches that can enhance their performance to microbial degradation [14]. Moreover, although hemp fibers have the potential to be used by different industries, hemp stumps are typically discarded as agro-waste [6,9]. In this sense, utilizing hemp agro-waste (HW) as a raw material for the industry has been shown to be extremely important and effective, particularly in terms of environmental and economic benefits, adopting circularity as the driving force [6,9].

Hemp bast is chemically composed of cellulose (70.2–74.4 wt%), hemicellulose (17.9–22.4 wt%), lignin (3.7–5.7 wt%), and gums that include pectin (2–4 wt%), and fat and wax (1–2 wt%) [14,15]. In this way, the transformation of HW into cellulosic and non-cellulosic biopolymer materials is a sustainable and convenient way to exploit waste and address current environmental and economic challenges [1,8].

There are several methods used for the recovery of gums, lignin, and other non-cellulosic components from hemp fibers, and among them are the chemical treatments that use organic and inorganic acids, peroxide, chelating agents, sodium chlorite, sodium sulfite, and anhydrides [1,2,7,14]. However, chemical treatments can cause several negative impacts on the environment, producing effluents with large load pollutants and high energy consumption, which increases production costs [1,2]. In addition, traditional heating methods that require gas, electricity, steam, and other energy sources for the hemp degumming exhibit several disadvantages, such as long treatment times to reach the required temperature, high operational costs, and process inefficiency [1]. Thus, given the growing need to develop more ecological and sustainable practices, ionic liquids (ILs), a recent class of organic salts, have revealed promising properties to be used as green solvents [1]. Furthermore, ILs have been used to dissolve and process biopolymers such as silk, chitin, chitosan, wood, lignocellulose, and cellulose [2,16,17,18,19,20]. However, ILs have a series of limitations, including toxicity, a high cost, and a destructive structural impact on cellulose, which limits their use when it is intended to produce cellulose-based materials from agro-industrial wastes [1]. Therefore, as a substitute for ILs, deep eutectic solvents (DESs) have emerged as a new type of sustainable green solvents. DESs are composed of two or more components, forming eutectic mixtures that have a lower eutectic point temperature in comparison with an ideal liquid mixture [1,21,22]. Additionally, DESs have some notable advantages over ILs, such as lower cost, recyclability, excellent biodegradability, and low toxicity, which makes them more attractive for treating hemp fibers [1,8,21,23]. Moreover, some types of DESs, like choline chloride (ChCl) and urea in a 1:2 mol ratio, have already been studied for degumming hemp fibers without modifying their cellulosic structure [1,8]. In addition, the use of DESs as solvents and/or catalysts in organic reactions, in biotransformations, and both in cosmetic and pharmaceutical applications has received significant attention in recent years [24,25,26]. Additionally, DESs have increased the range of possibilities for dissolving and modifying several biopolymers, such as cellulose, lignin, and chitin [27,28,29]. However, most studies that use HW focused only on the dissolution and extraction of their biopolymers with DESs, which limits their use as a sustainable approach for the development of new materials [21]. Therefore, it is necessary to develop new strategies to reuse the hemp biopolymers dissolved into the DESs, namely gummy compounds that include pectin, oil, wax, minerals, lignin, and other non-cellulosic components in order to find more ecological processing solutions for producing functional and bioactive materials with added value [1,21].

Electrospinning is a simple, versatile, and promising technique that allows the processing of different biopolymers into nanofiber-based polymeric structures [2,21,30,31]. Furthermore, the polymers used in electrospinning can impart unique properties to the materials, namely magnetic, optical, electrical, and antimicrobial properties, which are useful for a wide range of applications [1,21]. Nonetheless, electrospinning frequently requires the use of volatile organic solvents to dissolve biopolymers and obtain electrospinning solutions. However, although organic solvents volatilize during the electrospinning process to form nanofibers, these solvents are generally harmful to the environment and human health [21,31]. In this way, the suitable solubility and viscosity of the biopolymers in DESs could benefit the electrospinning process and should be seen as a sustainable alternative that overcomes the limitations of the organic solvents and even the ILs.

Hence, this research study opens a perspective of using HW, namely the gel-based gums, lignin, and other non-cellulosic components, efficiently solubilized in DESs to produce composite nanofibers through electrospinning. For this purpose, the biopolymers obtained from HW dissolved in DESs were combined with the Poly(vinyl alcohol) (PVA), a water-soluble, semicrystalline, totally biodegradable, non-toxic, and biocompatible polymer, and the gel-based blends formed were processed into composite nanofibers using a needle-free electrospinning technique based on a rotating roller electrode, due to its ability to enable a higher production rate in comparison to the conventional needle electrospinning [21,32]. Therefore, it is worth noticing that, to the best of our knowledge, this is the first time that HW is successfully electrospun into nanofibers applying directly the gel-based HW biopolymers dissolved in ChCl:Glycerol (1:2) and ChCl:Urea (1:2) DESs.

## 2. Results and Discussion

### 2.1. Attenuated Total Reflectance-Fourier Transform Infrared Spectroscopy (ATR-FTIR) Analysis

In recent years, finding solutions to convert waste into new materials has become the main target of different studies [7,33,34]. In addition, processing new materials through electrospinning using green solvents or water-soluble polymers has proven to be an effective approach to overcome the drawbacks of organic solvents used for polymer dissolution in the electrospinning process and a sustainable alternative to conventional non-biodegradable petroleum-based polymers [7,21,35,36].

Concerning that, in this study, the gums, lignin, and other non-cellulosic components in HW were first recycled using ChCl:Glycerol (1:2) and ChCl:Urea (1:2) DESs and ultrasound in order to promote the dissolution of the hemp constituents in DES mixtures. Then, 0.5%, 2.0%, and 4.0% (*v*/*v*) of the gel-based HW biopolymers dissolved into the DESs were blended with the PVA and used directly in the electrospinning to fabricate the electrospun composite nanofibers.

**Figure 1:**
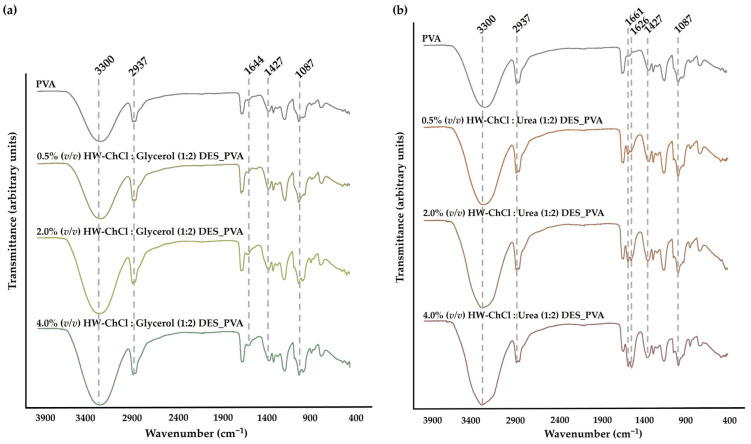
ATR-FTIR analysis of the produced composite nanofibers and of the PVA raw material: gel-based blends of hemp agro-waste (HW) dissolved in ChCl:Glycerol (1:2) (**a**) and in ChCl:Urea (1:2) (**b**) DESs and PVA.

The chemical composition of the produced HW-DESs_PVA composite nanofibers was then analyzed by ATR-FTIR. The Figure 1 reports the FTIR spectra of the raw PVA nanofibers, and the HW dissolved in the ChCl:Glycerol (1:2) and ChCl:Urea (1:2) DES mixtures combined with PVA, e.g., HW-ChCl:Glycerol (1:2) DES_PVA and HW-ChCl:Urea (1:2) DES_PVA, respectively.

In the PVA spectrum, a significant peak at the wavenumbers of 3300 cm^−1^ was related to the -OH stretching vibration. The peak at 2937 cm^−1^ belongs to the stretching vibration of the CH bond, while the peaks at 1427 cm^−1^ and 1087 cm^−1^ concern the CH_2_ bending and C-O stretching bands, respectively, Figure 1a,b [32,36].

In turn, the major peaks related to PVA were evident in the spectra corresponding to the composite nanofibers produced from gel-based blends of HW dissolved in DESs and PVA (HW-DESs_PVA) (see Figure 1a,b for further details). Moreover, the characteristic peaks at 1644 cm^−1^, 1661 cm^−1^, and 1626 cm^−1^, typically associated with the vibration absorption of the aromatic ring in lignin molecules, were observed in the spectrum of the PVA nanofibers combined with the HW’s gums, lignin, and other non-cellulosic components dissolved into ChCl:Glycerol (1:2) and ChCl:Urea (1:2) DESs, being more evidenced by nanofibers containing the higher content of the HW dissolved in the DES mixtures, Figure 1a,b [8]. Nevertheless, it is more likely that these peaks are associated with the DES components trapped in the nanofiber due to the 16:1 (*w*/*w*) ratio of DES to HW used during the extraction process. Additionally, the characteristic peak associated with the stretching vibration of C-H bonds in gums at 2975 cm^−1^ was not detected in the spectrum [8]. This absence is attributed to the overlap of HW’s characteristic peaks with the bands of the PVA, the main component of the nanofibers, as well as with the functional groups of DES mixtures, since only 28.02% (*w*/*w*) and 28.56% (*w*/*w*) of HW were dissolved in the ChCl:Glycerol (1:2) and ChCl:Urea (1:2) DESs, respectively, as determined from the recovered cellulosic part by filtration. However, a slight increase in the peak’s intensity, particularly at 3300 cm^−1^, was also observed when the dissolved HW in DESs rose from 0.5% (*v*/*v*) to 4.0% (*v*/*v*) in combination with the PVA, indicating that the incorporation of the HW did not change the chemical structure of the PVA. Therefore, the intensity of peaks related to PVA increases with the increase in the HW content, confirming the production of the electrospun composite nanofibers from gel-based blends of HW-DESs_PVA.

### 2.2. Characterization of the Morphologic Features and Static Image Analysis

PVA, a water-based synthetic polymer, has attracted great interest from researchers because of its excellent processability, low cost, biocompatibility, biodegradability, mechanical properties, and chemo-thermal stability [36]. In fact, according to previous studies, it is easy to prepare PVA electrospun nanofibers from different concentrations, and smooth beadless and random nanofibers with fiber diameters of 346.7 ± 123.7 nm have been obtained from 10% PVA (*w*/*v*) [36].

**Figure 2:**
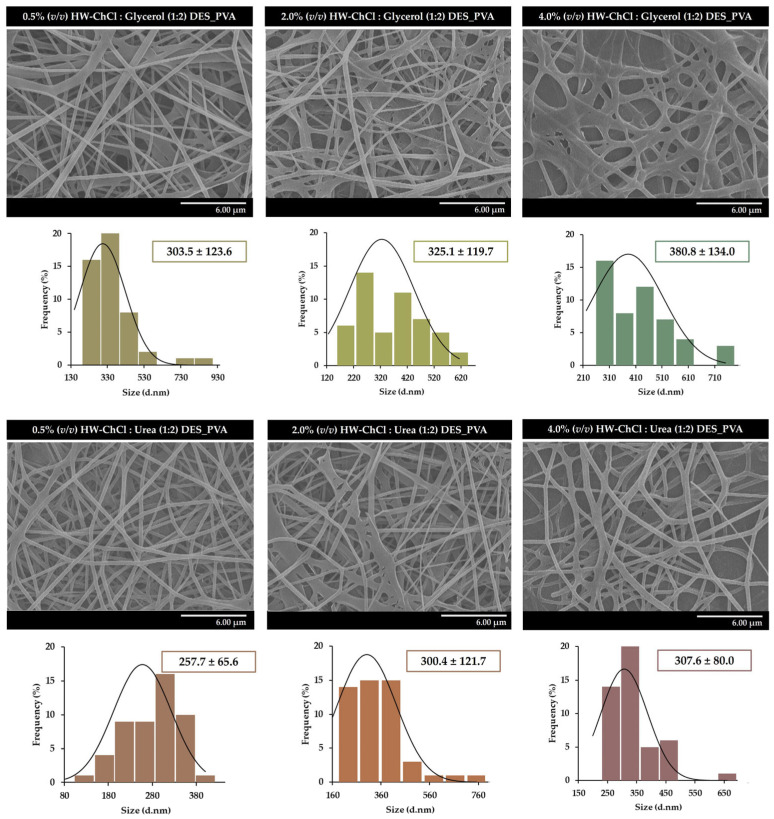
Characterization of the morphological properties of the electrospun composite nanofibers produced from gel-based blends of HW-DESs_PVA using 0.5% (*v*/*v*), 2.0% (*v*/*v*), and 4.0% (*v*/*v*) of HW dissolved in ChCl:Glycerol (1:2) and ChCl:Urea (1:2) DESs and PVA.

In turn, although DESs are regarded as a promising alternative to conventional solvents for dissolving HW biopolymers, the gel-based biopolymers obtained from HW in DESs present challenges for electrospinning. Their high viscosity and conductivity can hinder the formation of a stable jet, impacting the electrospinning process. Thus, to overcome these limitations, 10% PVA (*w*/*v*) was added to convert HW into new composite nanofibers through electrospinning. For that purpose, the gel-based blends of HW-DESs_PVA were used directly in the electrospinning under the same processing conditions. The surface morphology of the composite nanofibers was characterized by SEM and the diameters of the fibers were determined using the ImageJ software v2.3.0. Figure 2 presents the SEM images of the nanofibers obtained from 0.5%, 2.0%, and 4.0% (*v*/*v*) HW-DESs_PVA, respectively.

The SEM images show that it is possible to obtain uniform electrospun nanofibers, without the presence of beads, from HW dissolved in DESs, Figure 2. In addition, the size measurements reveal that the composite nanofibers produced from the gel-based blend with 0.5% (*v*/*v*) HW-ChCl:Glycerol (1:2) DES_PVA exhibit an average diameter of 303.5 ± 123.6 nm, while the 2.0% (*v*/*v*) and 4.0% (*v*/*v*) HW-ChCl:Glycerol (1:2) DES_PVA nanofibers display a mean diameter of 325.1 ± 119.7 nm and 380.8 ± 134.0 nm, respectively.

The slight increase in the nanofibers’ mean diameter can be explained by the fact that the viscosity of the gel-based HW dissolved in the DESs increases the viscosity of the DES solution, and consequently, the same happens when the ratio of the HW dissolved in the ChCl:Glycerol (1:2) DES was increased in the electrospinning solution of 0.5% (*v*/*v*) to 4.0% (*v*/*v*). Thus, by increasing the concentration of the electrospinning solution, the viscosity of the solution increases, as well as the polymer chain entanglements, resulting in higher fiber diameters.

A similar effect was observed for the ChCl:Urea (1:2) DES, with the 0.5% (*v*/*v*) HW-ChCl:Urea (1:2) DES_PVA nanofibers showing fibers with an average diameter of 257.72 ± 65.63, slightly smaller than the diameter of the 2.0% (*v*/*v*) and 4.0% (*v*/*v*) HW-ChCl:Urea (1:2) DES_PVA nanofibers (e.g., 300.4 ± 121.7 nm and 307.6 ± 80.0 nm, respectively).

Similarly, Rong et al. synthesized lignin fiber aerogels via electrospinning from commercial dealkaline lignin, as the lignin resource, and PVA, dissolved in choline chloride–lactic acid (ChCl–LA) (1:2) DES [21]. The obtained results demonstrated that the commercial dealkaline lignin increased the viscosity of the ChCl–LA (1:2) DES solution, compromising the quality of lignin fibers. In the same way, the dissolution of PVA increased the viscosity of the DES solution [21]. In addition, the lignin fiber aerogels composed of lignin nanofibers exhibited diameters ranging from 320 to 480 nm, similar to those presented in the present study.

### 2.3. Characterization of the Mechanical Properties of the Electrospun Composite Nanofibers

The electrospun composite nanofibers produced from gel-based blends of HW-DESs_PVA must display mechanical properties that allow them to provide suitable mechanical strength and support. In this context, the mechanical characterization of the electrospun nanofibers produced from gel-based HW dissolved in the DES mixtures and PVA was performed through the tensile assay by measuring the tensile strength, elongation at break, and Young’s modulus, as summarized in Table 1.

Regarding the raw PVA nanofibers, the obtained values for tensile strength, elongation at break, and Young’s modulus were 4.43 ± 1.84 MPa, 9.21 ± 4.51%, and 42.42 ± 13.29 MPa, respectively. In turn, the electrospun nanofibers containing a higher content of the HW presented a superior tensile strength, reaching a value of 12.70 ± 0.57 MPa for the 4.0% (*v*/*v*) HW-ChCl:Glycerol (1:2) DES_PVA and 14.16 ± 0.99 MPa for the 4.0% (*v*/*v*) HW-ChCl:Urea (1:2) DES_PVA, respectively, as shown in Table 1. Moreover, Young’s modulus also improved when the HW was incorporated into PVA, reaching a maximum of 257.07 ± 15.46 MPa for the 4.0% (*v*/*v*) HW-ChCl:Glycerol (1:2) DES_PVA and 285.56 ± 34.51 MPa for the 4.0% (*v*/*v*) HW-ChCl:Urea (1:2) DES_PVA, respectively. In addition, the experimental data show that the elongation at break slightly decreased with the addition of the gel-based HW dissolved in the DESs from 9.21 ± 4.51% to 5.62 ± 0.05% for the 0.5% (*v*/*v*) HW-ChCl:Glycerol (1:2) DES_PVA, 4.27 ± 0.64% for the 2.0% (*v*/*v*) HW-ChCl:Glycerol (1:2) DES_PVA, and 4.94 ± 0.08% for the 4.0% (*v*/*v*) HW-ChCl:Glycerol (1:2) DES_PVA, respectively. A similar effect on the elongation at break of the HW-ChCl:Urea (1:2) DES_PVA nanofibers was observed when the gel-based HW dissolved into ChCl:Urea (1:2) DES was added to the PVA, reaching values of 5.13 ± 0.93% for the 0.5% (*v*/*v*) HW-ChCl:Urea (1:2) DES_PVA, 5.29 ± 1.04% for the 2.0% (*v*/*v*) HW-ChCl:Urea (1:2) DES_PVA, and 4.99 ± 0.38% for the 4.0% (*v*/*v*) HW-ChCl:Urea (1:2) DES_PVA, respectively. Therefore, these results suggested an enhancement in both tensile strength and Young’s modulus, which is caused due to the formation of strong hydrogen bonds between the recycling HW biopolymers, like the lignin, and the PVA. Moreover, when the lignin biopolymer is combined with the PVA, the lignin can act as a cross-linker due to its low ductility, resulting in a slight decrease in the elongation at break. Additionally, lignin can polymerize and act as a binder to other cellulosic compounds [37,38].

In a similar way, Korbag et al. prepared PVA/lignin films and they recorded that the tensile strength of PVA/lignin films increased from 19.12 MPa to 38.44 MPa, while the elongation at break slightly decreased from 266.50% to 213.9%, respectively [38]. Furthermore, the Young’s modulus also increased to 83.22 MPa, in comparison to the raw PVA [38].

### 2.4. Characterization of the Optical Properties of the Produced Electrospun Composite Nanofibers

The chromaticity of the composite nanofibers produced from gel-based blends of HW-DESs_PVA through electrospinning was evaluated according to the CIELAB color system, and the main data were detailed in Table 2. The raw PVA showed a higher whiteness (WI = 98.01), which slightly decreased when the gel-based HW dissolved in the DESs was added. However, the WI values did not demonstrate any significant difference in relation to the raw PVA, the control group. In addition, the incorporation of the HW dissolved in both ChCl:Glycerol (1:2) and ChCl:Urea (1:2) DES mixtures did not provide a significant color difference in the nanofibers, leading only to a slight decrease in red (a*) and increase in blue (b*) with values close to zero. Therefore, a positive b* value indicative of the yellowing of the nanofibers was not observed. Such a trend could be expected due to the yellowish aspect of the hemp gums, lignin, and other non-cellulosic substances dissolved in the DESs.

Moreover, the brightness parameter (L*), with values ranging from 0 (absolute black) to 100 (absolute white), was high (above 90.00) for all samples, which indicates the whiteness of the electrospun composite nanofibers produced from gel-based blends of HW-DESs_PVA. The L* value was superior to the raw PVA nanofibers (L* = 98.06 ± 0.01) and lowest for the nanofibers composed by 4.0% (*v*/*v*) HW-ChCl:Urea (1:2) DES_PVA (L* = 92.26 ± 0.01), which was more difficult to electrospin and consequently revealed an inferior thickness.

In this way, the CIELab color parameters (L*, a*, and b*) based on human perception of colors indicated the whiteness of the prepared nanofibers. Furthermore, the reported chromaticity data showed that the gel-based HW dissolved in the DESs only produced a slight difference in the ΔE* values, Table 2.

In fact, Viscusi et al. previously obtained electrospun polyamide fibers reinforced with hemp fibers dissolved in 1-ethyl-3-methylimidazolium dicyanamide IL [2]. The chromaticity data suggested that the incorporation of hemp fibers in the IL reduced the whiteness of the polyamide, resulting in a yellowish appearance. Such a result is in agreement with the increase in yellowness (b*), which resulted in a higher ΔE* value [2].

### 2.5. Evaluation of the Antioxidant Activity of the Produced Electrospun Composite Nanofibers

The biopolymers obtained from HW, as well as DES mixtures, present relevant bioactive properties, namely antioxidant capacity, which was tested using the ABTS radical-scavenging measurement method. This assay exploits the fact that ABTS free radicals become stable and change their blue color to colorless by accepting a hydrogen ion from the antioxidant [36]. Hence, in this study, the scavenging ability of the electrospun composite nanofibers produced from gel-based blends of HW dissolved in DESs and PVA was determined to evaluate the effect of adding the gel-based gums, lignin, and other non-cellulosic components of HW dissolved in ChCl:Glycerol (1:2) and ChCl:Urea (1:2) DESs to the PVA on the antioxidant properties.

**Figure 3:**
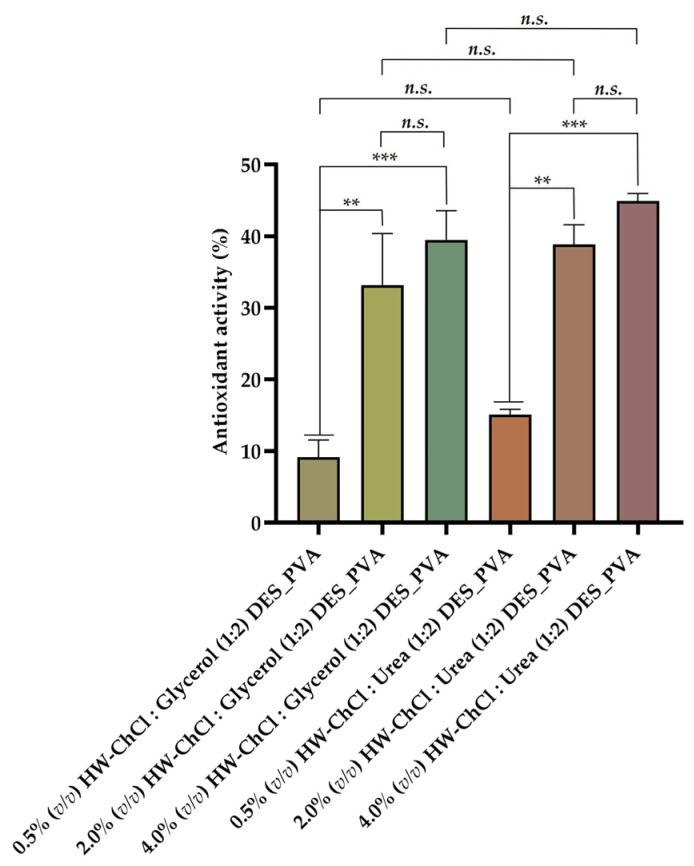
Antioxidant activity evaluation of the electrospun composite nanofibers prepared from gel-based blends of HW-DESs_PVA using HW dissolved in ChCl:Glycerol (1:2) and in ChCl:Urea (1:2) DESs and PVA (Data are presented as the mean ± standard deviation, *n.s. p* > 0.05, ** p < 0.01 and *** p < 0.001).

Figure 3 show that the electrospun nanofibers composed by 0.5% (*v*/*v*) HW-DESs_PVA exhibit a reduced antioxidant activity (4.27 ± 8.62% for 0.5% (*v*/*v*) HW-ChCl:Glycerol (1:2) DES_PVA) and 9.98 ± 8.86% (for 0.5% (*v*/*v*) HW-ChCl:Urea (1:2) DES_PVA), while the electrospun nanofibers produced from 4.0% (*v*/*v*) HW-DESs_PVA presented the highest antioxidant ability (39.49 ± 4.09% for 4.0% (*v*/*v*) HW-ChCl:Glycerol (1:2) DES_PVA) and 44.87 ± 1.08% for 4.0% (*v*/*v*) HW-ChCl:Urea (1:2) DES_PVA). Thus, the increased content of the HW dissolved in the DESs incorporated into the nanofibers resulted in a higher activity to scavenge ABTS free radicals, which was attributed to the hydroxyl and carbonyl groups present in the structure of the biopolymers constituents of the HW, as well as in the components of the DES mixtures. Therefore, the nanofibers produced from 4.0% (*v*/*v*) HW dissolved in DESs showed a powerful and effective antioxidant potential, since there are more hydroxyl and carbonyl groups available to react with the free radicals, such as ABTS, leading to an increase in their scavenging.

Such results align with those obtained in another study reported in the literature by Viscusi et al., wherein hemp fibers dissolved in an ionic liquid were incorporated into polyamide 6 [2]. The obtained data reveal an enhancement in the antioxidant activity of the composite electrospun membranes by 21% to 89%, which suggests that hemp fibers possess the ability to scavenge DPPH free radicals due to the presence of hydroxyl groups in the chemical structure of the natural fibers, such as cellulose, hemicellulose, and lignin [2]. Hence, the presence of hydroxyl groups in polysaccharides contributes to their antioxidant properties, since these groups have the ability to scavenge free radicals by donating hydrogen atoms, potentially demonstrating an antioxidant effect [2,39].

Moreover, ChCl:Glycerol-based DES has been reported as suitable for extracting bioactive compounds with antioxidant activity from wastes [40]. Similarly, Pal et al. successfully used a DES mixture composed of ChCl:Urea to extract polyphenolic antioxidants from biomass waste-derived onion peel [41].

### 2.6. Evaluation of the Antimicrobial Activity of the Produced Electrospun Composite Nanofibers

**Figure 4:**
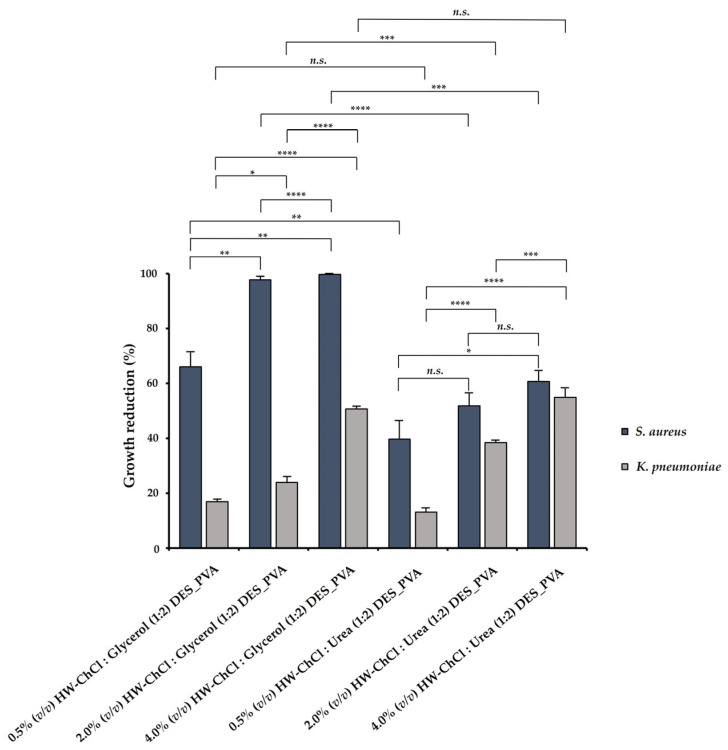
Evaluation of the antibacterial efficiency toward *Staphylococcus aureus* (*S. aureus*) and *Klebsiella pneumoniae* (*K. pneumoniae*) of the produced electrospun composite nanofibers prepared from gel-based blends of HW-DESs_PVA using HW dissolved in ChCl:Glycerol (1:2) and in ChCl:Urea (1:2) DESs and PVA (Data are presented as the mean ± standard deviation, *n.s. p* > 0.05, * p < 0.05, ** p < 0.01, *** p < 0.001, and **** p < 0.0001).

In this study, the antibacterial ability of the electrospun composite nanofibers produced from gel-based blends of HW dissolved in DES mixtures and PVA was evaluated against *Staphylococcus aureus* (*S. aureus*) and *Klebsiella pneumoniae* (*K. pneumoniae*), and the percentages of reduction in bacterial growth are shown in Figure 4. According to the obtained results, the electrospun HW-DESs_PVA nanofibers were most effective against *S. aureus* (gram-positive bacteria), since the cell wall of gram-negative bacteria, like *K. pneumoniae*, present a complex multilayered structure and a higher thickness. Indeed, other studies indicate that the susceptibility of the gram-positive and gram-negative bacteria to antimicrobial agents could be attributed to variations in the structure and composition of the cytoplasmatic membrane and/or outer membrane cell wall [42].

In addition, the higher content of HW dissolved into the DES mixtures induced the largest growth inhibition, displaying an inhibitory effect in *S. aureus* growth of 99.72 ± 0.05% for the 4.0% (*v*/*v*) HW-ChCl:Glycerol (1:2) DES_PVA nanofibers and 60.68 ± 4.10% for the 4.0% (*v*/*v*) HW-ChCl:Urea (1:2) DES_PVA nanofibers, respectively. Similarly, the 4.0% (*v*/*v*) HW-DESs_PVA nanofibers enabled a higher inhibition of the *K. pneumoniae* growth, reaching a value of 50.65 ± 1.14% for the nanofiber produced from the HW dissolved in ChCl:Glycerol (1:2) DES and 54.94 ± 3.42% in ChCl:Urea (1:2) DES, respectively. Moreover, these data showed that the incorporation of the HW dissolved into ChCl:Glycerol (1:2) DES in the electrospun nanofibers exerted a superior inhibitory effect in bacterial growth in comparison with the HW dissolved in the ChCl:Urea (1:2) DES. Therefore, it can be concluded that, among the tested DES mixtures, the ChCl:Glycerol (1:2) was the most efficient for the extraction of antimicrobial compounds from the HW.

In the literature, it has been described that hemp biopolymers, like lignin, have antibacterial activity associated with the presence of phenolic hydroxyl methoxy groups, which can damage the bacteria cell membrane, causing the bacteria lysis [43,44]. Moreover, more recently, ChCl-based natural DES extracted from phenolic compounds showed a significant antibacterial activity against several gram-positive and gram-negative bacteria [45].

## 3. Conclusions

In this study, we proposed the manufacturing of new gel-based blends to produce composite nanofibers from PVA and HW dissolved in DESs, as a greener endeavor toward waste management. The gums, lignin, and other non-cellulosic components in HW were successfully recovered with ChCl:Glycerol (1:2) and ChCl:Urea (1:2) DESs using an ultrasound-assisted extraction method, and then they were directly blended with PVA in ratios of 0.5%, 2.0%, and 4.0% (*v*/*v*) for subsequent electrospinning into nanofibers. The compatibility between the components of the mixtures, namely of PVA and the HW dissolved in DESs, was proven through ATR-FTIR analysis. Moreover, the obtained electrospun nanofibers were characterized in terms of surface morphology by SEM and the nanofibers presented uniform morphology without the presence of beads, with fiber diameters ranging between 257.7 ± 65.6 nm and 380.8 ± 134.0 nm, respectively.

From the point of view of the mechanical properties, a notable Young’s modulus and tensile strength were observed, giving the material a large potential of applications. Furthermore, in terms of optical properties, the nanofibers exhibited a WI above 90%, thus confirming that the presence of the lignin and other components recovered from the HW using the DES mixtures did not provide for the nanofibers a yellow appearance. Additionally, the electrospun membranes confirmed that the incorporation of the HW dissolved in DESs imprinted antioxidant and antibacterial properties to the nanofibers, which are essential for their application in different fields.

Therefore, considering the obtained results, the electrospun composite nanofibers were successfully fabricated from gel-based blends of HW biopolymers and PVA in a bid to reduce waste and promote sustainability and environmental awareness. Moreover, taking advantage of their distinctive properties, such as biocompatibility, biodegradability, natural absorbency, mechanical strength, durability, and eco-friendly nature, the electrospun HW-DESs_PVA composite nanofibers offer versatile applications across several industries. These innovative nanofibers provide sustainable solutions for various purposes, including wound dressings, absorbent materials for water treatment and air filtration, biodegradable packaging, and reinforcement in textiles. Notably, they stand out when compared to counterparts sourced from other plant sources, like kenaf and jute.

For future work, the authors propose estimating the amount of HW-DESs incorporated into the produced electrospun composite nanofibers by employing the gel-based blends of HW-DESs_PVA. To achieve this, a comprehensive analysis of the solubility and deconstruction of DES in water, along with its interaction with PVA during blending is essential. This analysis should encompass the utilization of analytical techniques for quantification and conducting experimental validations to achieve the most accurate estimation.

In addition, although HW has been explored as a source of non-cellulosic components, the cellulosic part of HW after degumming with the DESs can be also explored to develop new hemp cellulosic-based materials, without the need of the retting procedure, embracing in a zero-waste approach. Additionally, the potential recyclability of DESs for extracting lignin and other non-cellulosic components from HW will undergo multiple assessments. These evaluations aim to verify process reliability and enhance the pathway toward a more sustainable future.

## 4. Materials and Methods

### 4.1. Materials

Hemp agro-waste (HW) was supplied by local producers (Fundão region, Portugal). Urea, Nutrient Agar (NA), Nutrient Broth (NB), Tween 80, and sodium chloride (NaCl) were acquired from Sigma-Aldrich (Sigma-Aldrich, St. Louis, MO, USA). Choline chloride 99% (ChCl) and glycerol were purchased from Fisher Scientific (Fisher Scientific, Leicestershire, UK). Poly(vinyl alcohol) PVA (115,000 g/mol) was provided from VWR Chemicals (VWR Chemicals, Leuven, Belgium). ABTS was acquired from Panreac (Panreac, Barcelona, Spain). Potassium persulfate was supplied from Acros Organics (Acros Organics, Geel, Belgium). Phosphate-buffered saline (PBS) (pH 7.4) was purchased from Alfa Aesar (Ward Hill, MA, USA). *S. aureus* (ATCC 6538) and *K. pneumoniae* (ATCC 4352) were purchased from the American Type Culture Collection (Manassas, VA, USA).

### 4.2. Preparation of DESs and Dissolution of HW Using DESs

Firstly, the ChCl:Glycerol DES was formed under stirring blending the ChCl and Glycerol at a molar ratio of 1:2. The resulting mixture was heated at 80 °C until a clear and homogeneous colorless liquid was obtained. Similarly, the ChCl:Urea DES was generated by combining ChCl and Urea in a 1:2 molar ratio, followed by continuous magnetic stirring at 80 °C until a transparent liquid was formed. Then, the HW was washed with water to remove the superficial impurities and dried in an oven. After that, the hemp fibers were ground into powder by a high-speed crusher and submerged into the DES mixtures in lab flasks in a DES to HW powder ratio of 16:1 (*w:w*). The lab flasks were sonicated at 70 °C using an ultrasound bath with distilled water for 15 h. Afterward, the mixtures were subjected to vacuum filtration, and the filtrate composed of the gel-based gums, lignin, and other non-cellulosic substances was recovered and used as a biopolymer source for manufacturing composite nanofibers via electrospinning, while the solid cellulosic part was stored for later studies.

### 4.3. Preparation of the Electrospinning Polymeric Solutions

The gel-based gums, lignin, and other non-cellulosic components obtained after the HW dissolution in DESs were directly used in the electrospinning. Moreover, PVA was dissolved in distilled water at 10% (*w*/*v*), under constant stirring, at 90 °C until to form a uniform solution. After that, the HW dissolved in DESs were blended with PVA at different ratios (0.5% (*v*/*v*), 2.0% (*v*/*v*), and 4.0% (*v*/*v*)), and stirred at room temperature for 1 h before electrospinning to form the electrospun gel-based blends.

### 4.4. Electrospinning of the Composite Nanofibers

Electrospinning was performed using a needle-free Nanospider™ machine (Nanospider laboratory machine NS LAB 500S from Elmarco s.r.o., Liberec, Czech Republic, http://www.elmarco.com, accessed on 18 October 2023) and a polypropylene nonwoven fabric (purchased from ELmarco) to collect the electrospun HW-DESs_PVA nanofibers. The electrospinning parameters of each gel-based polymeric blend solution were set to an applied voltage of 80 kV, maintaining a working distance of 13 cm between the electrode and the collector, and employing an electrode rotation rate of 55 Hz. The electrospinning process was conducted at 25 °C. Additionally, the raw PVA nanofibers were produced for comparative purposes.

### 4.5. Attenuated Total Reflectance-Fourier Transform Infrared Spectroscopy (ATR-FTIR) Analysis

The ATR-FTIR spectra for the electrospun composite nanofibers produced from gel-based blends of HW-DESs_PVA were captured using a Thermo Nicolet is10 FTIR spectrometer (Thermo Nicolet is10 FTIR Spectrophotometer, Waltham, MA, USA). The data were collected using a spectral bandwidth ranging from 400 to 4000 cm^−1^, maintaining a spectral resolution of 4 cm^−1^, and an average of 64 scans. Moreover, the spectrum of the raw PVA was recorded for comparative analysis.

### 4.6. Characterization of the Morphologic Features of the Produced Electrospun Composite Nanofibers

The Hitachi S-3400N Scanning Electron Microscope (Hitachi, Tokyo, Japan) was used to examine the surface morphology of the electrospun composite nanofibers fabricated from gel-based blends of HW-DESs_PVA. For SEM observation, the electrospun nanofibers were previously cut into small sections, affixed onto aluminum stubs using Araldite glue, and then received a thin gold layer through an Emitech K550 sputter coater (Emitech Ltd., Ashford, UK) to heighten image contrast. To maintain consistency, all the samples were simultaneously sputtering coating for the same period of time, aiming to minimize variations introduced during this process. The SEM images were captured at a magnification of ×5000 and an accelerating voltage of 20 kV.

### 4.7. Static Image Analysis of the Produced Electrospun Composite Nanofibers

The obtained SEM images were used to measure the fiber diameters through the ImageJ processing software (ImageJ, National Institutes of Health, Bethesda, MD, USA) [46]. Afterwards, the diameter distribution of the electrospun composite nanofibers produced from gel-based blends of HW-DESs_PVA was further evaluated using the GraphPad Prism 6 software (Prism Software, La Jolla, CA, USA).

### 4.8. Characterization of the Mechanical Properties of the Produced Electrospun Composite Nanofibers

The mechanical characteristics of the electrospun composite nanofibers produced from gel-based HW-DESs_PVA were evaluated at room temperature using a tensile test machine (DY-35, Adamel Lhomargy, Roissy en Brie, France) connected to a 100 N static load cell, following to the guidelines set by the Standard Test Method for Tensile Properties of Polymer Matrix Composite Materials (ASTM standard D3039/D3039 M) [47]. Prior to the evaluation, specimens with a gauge length of 6 cm and a width of 2 cm were prepared and positioned vertically between the clamps. The distance between the clamps was set to 2 cm and the deformation rate maintained at 2 mm/min. The parameters of interest, namely the tensile strength, Young’s modulus, and elongation at break, were determined from the average of at least three independent measurements.

### 4.9. Characterization of the Optical Properties of the Produced Electrospun Composite Nanofibers

The color difference (Δ*E*) between the raw PVA nanofibers and the electrospun composite nanofibers fabricated from gel-based blends of HW-DESs_PVA was determined by using a Datacolor 110 spectrophotometer (Datacolor company, USA) with a D65 standard illuminant from CIELab values (L*, a*, and b*) and calculated according to Equation (1):

(1) $${\Delta E}^{*\;}=\sqrt{{{(L_{2}}^{*}-{L_{1}}^{*})}^{2}+{{(a_{2}}^{*}-{a_{1}}^{*})}^{2}+{{(b_{2}}^{*}-{b_{1}}^{*})}^{2}}$$

where L* represents darkness to lightness, with 0 being a perfect black and 100 being a perfect white, and a* and b* the chromaticity coordinates (a*—redness: green to red and b*—yellowness: blue to yellow, respectively).

Moreover, the Whiteness index (*WI*) of the samples was estimated through Equation (2):

(2) $$WI=100-\sqrt{{\left(100-L^{*}\right)}^{2}+{(a}^{*2}+b^{*2})}$$

For each sample, three measurements were made at random points, and the data were reported as the average of these independent determinations.

### 4.10. Evaluation of the Antioxidant Activity of the Produced Electrospun Composite Nanofibers

The electrospun composite nanofibers produced from gel-based blends of HW-DESs_PVA were assessed for their antioxidant activity using the ABTS radical decolorization assay. Initially, ABTS radical (ABTS+) cation was produced by reacting 5 mL of ABTS (7 mM) stock solution with 88 µL of 2.4 mM potassium persulfate. The mixture was then incubated in the darkness at room temperature during 12–16 h. Before starting the assay, the ABTS+ solution was diluted to an absorbance of 0.700 ± 0.025 at 734 nm with phosphate buffer saline (0.1 M, pH 7.4). Subsequently, 10 mg of each sample was incubated with 10 mL of ABTS+ solution in dark for 30 min, and the scavenging capability of ABTS+ at 734 nm was estimated through Equation (3):

(3) $$Antioxidant\;activity\;(\%)=\frac{A_{control}-A_{sample}}{A_{control}}\times 100$$

where *A_control_* represents the absorbance of the remaining concentration of ABTS+ in the control sample (e.g., raw PVA nanofibers) and *A_sample_* is the absorbance of the remaining ABTS+ concentration in the presence of the HW-DESs_PVA nanofibers. All experiments were conducted in triplicate.

### 4.11. Evaluation of the Antimicrobial Activity of the Produced Composite Nanofibers

The ability of the electrospun composite nanofibers fabricated from gel-based blends of HW-DESs_PVA to inhibit bacteria growth was assessed by exposing them to *S. aureus* (ATCC 6538) and *K. pneumoniae* (ATCC 4352), following the Japanese Industrial Standard JIS L 1902:2002 [48]. To conduct the assessment, bacterial suspensions were prepared at a concentration of 1 × 10^5^ CFU/mL by transferring colonies from overnight cultures in sterile NB medium onto the samples. After that, the samples were examined immediately after the inoculation (*T_0h_*) and following 18–24 h of contact with the inoculum at 37 °C (*T_24h_*). Subsequently, each sample was vigorously vortex-mixing for 30 s with 0.85 (*w*/*v*) NaCl and 2 mL/L of Tween 80. Next, serial dilutions were prepared with 0.85 (*w*/*v*) NaCl and plated on NA plates. After overnight incubation at 37 °C, the bacterial colonies were counted and expressed as CFU/mL. The percentage of bacterial growth inhibition was estimated using Equation (4):

(4) $$Antimicrobial\;activity\;(\%)=\frac{C-S}{C}\times 100$$

where *C* stands for the CFU/mL calculated for the control group (e.g., raw PVA nanofibers) while *S* represents the electrospun HW-DESs_PVA nanofibers, respectively.

### 4.12. Statistical Analysis

The statistical analysis of the data was performed utilizing GraphPad Prism 6 software (GraphPad Software, La Jolla, CA, USA) through a one-way analysis of variance (ANOVA), followed by Tukey’s multiple comparison test. Statistical significance was determined at a significance level of *p* < 0.05. All experiments were conducted in triplicate and the results were presented as the mean value ± standard deviation (SD).

## Figures and Tables

**Table 1:** The tensile strength, elongation at break, and Young’s modulus of the electrospun composite nanofibers produced from gel-based blends of HW-DESs_PVA using HW dissolved in ChCl:Glycerol (1:2) (a) and in ChCl:Urea (1:2) DESs and PVA.

	Thickness (mm)	Tensile Strength (MPa)	Young’s Modulus (MPa)	Elongation at Break (%)
PVA	0.36 ± 0.06	4.43 ± 1.84	42.42 ± 13.29	9.21 ± 4.51
0.5% (*v*/*v*) HW-ChCl:Glycerol (1:2) DES_PVA	0.23 ± 0.01	6.98 ± 0.33	124.17 ± 7.02	5.62 ± 0.05
2.0% (*v*/*v*) HW-ChCl:Glycerol (1:2) DES_PVA	0.16 ± 0.01	9.85 ± 1.00	232.21 ± 11.81	4.27 ± 0.64
4.0% (*v*/*v*) HW-ChCl:Glycerol (1:2) DES_PVA	0.14 ± 0.01	12.70 ± 0.57	257.07 ± 15.46	4.94 ± 0.08
0.5% (*v*/*v*) HW-ChCl:Urea (1:2) DES_PVA	0.18 ± 0.01	12.33 ± 1.20	254.18 ± 33.40	5.13 ± 0.93
2.0% (*v*/*v*) HW-ChCl:Urea (1:2) DES_PVA	0.15 ± 0.01	12.89 ± 1.17	246.95 ± 25.15	5.29 ± 1.04
4.0% (*v*/*v*) HW-ChCl:Urea (1:2) DES_PVA	0.12 ± 0.01	14.16 ± 0.99	285.56 ± 34.51	4.99 ± 0.38

**Table 2:** CIELAB parameters, color difference (ΔE*) values, and the whiteness index (*WI*) predicted for the raw PVA and the electrospun composite nanofibers produced from the gel-based blends of HW-DESs_PVA using HW dissolved in ChCl:Glycerol (1:2) (a) and in ChCl:Urea (1:2) DESs and PVA.

	L*	a*	b*	ΔE*	*WI*
PVA	98.06 ± 0.01	−0.11 ± 0.06	−0.43 ± 0.24	-	98.01
0.5% (*v*/*v*) HW-ChCl:Glycerol (1:2) DES_PVA	95.01 ± 0.24	−0.59 ± 0.01	−2.57 ± 0.02	3.76	94.35
2.0% (*v*/*v*) HW-ChCl:Glycerol (1:2) DES_PVA	95.61 ± 0.26	−0.40 ± 0.07	−0.96 ± 0.10	2.52	95.49
4.0% (*v*/*v*) HW-ChCl:Glycerol (1:2) DES_PVA	95.15 ± 0.86	−0.59 ± 0.04	−1.27 ± 0.04	3.07	94.95
0.5% (*v*/*v*) HW-ChCl:Urea (1:2) DES_PVA	96.84 ± 0.19	−0.36 ± 0.12	−0.69 ± 0.37	1.27	96.74
2.0% (*v*/*v*) HW-ChCl:Urea (1:2) DES_PVA	95.49 ± 0.70	−0.45 ± 0.07	−0.98 ± 0.11	2.65	95.36
4.0% (*v*/*v*) HW-ChCl:Urea (1:2) DES_PVA	92.26 ± 0.01	−1.34 ± 0.18	−2.60 ± 0.30	6.31	91.73

## Data Availability

Data that support the findings of this study are included in the article.
